# Shortened Hinge Design of Fab x sdAb-Fc Bispecific Antibodies Enhances Redirected T-Cell Killing of Tumor Cells

**DOI:** 10.3390/biom12101331

**Published:** 2022-09-21

**Authors:** Shuyu Huang, Aina Segués, Martin Waterfall, David Wright, Charlotte Vayssiere, Sander M. J. van Duijnhoven, Andrea van Elsas, Alice J. A. M. Sijts, Dietmar M. Zaiss

**Affiliations:** 1 Institute of Immunology and Infection Research, School of Biological Sciences, University of Edinburgh, Edinburgh EH9 3FL, UK; 2 Faculty of Veterinary Medicine, Department of Infectious Diseases and Immunology, Utrecht University, 3584 CS Utrecht, The Netherlands; 3 ImmunoPrecise Antibodies Ltd., 5349 AB Oss, The Netherlands; 4 Third Rock Ventures, San Francisco, CA 94158, USA; 5 Department of Immune Medicine, University Regensburg, 93053 Regensburg, Germany; 6 Institute of Clinical Chemistry and Laboratory Medicine, University Hospital Regensburg, 93053 Regensburg, Germany; 7 Institute of Pathology, University Regensburg, 93053 Regensburg, Germany

**Keywords:** bispecific antibody, cancer immunotherapy, mCD3E, mEGFR, hinge

## Abstract

T cell engager (TCE) antibodies have emerged as promising cancer therapeutics that link cytotoxic T-cells to tumor cells by simultaneously binding to CD3E on T-cells and to a tumor-associated antigen (TAA) expressed by tumor cells. We previously reported a novel bispecific format, the IgG-like Fab x sdAb-Fc (also known as half-IG_VH-h-CH2-CH3), combining a conventional antigen-binding fragment (Fab) with a single domain antibody (sdAb). Here, we evaluated this Fab x sdAb-Fc format as a T-cell redirecting bispecific antibody (TbsAbs) by targeting mEGFR on tumor cells and mCD3E on T cells. We focused our attention specifically on the hinge design of the sdAb arm of the bispecific antibody. Our data show that a TbsAb with a shorter hinge of 23 amino acids (TbsAb.short) showed a significantly better T cell redirected tumor cell elimination than the TbsAb with a longer, classical antibody hinge of 39 amino acids (TbsAb.long). Moreover, the TbsAb.short form mediated better T cell-tumor cell aggregation and increased CD69 and CD25 expression levels on T cells more than the TbsAb.long form. Taken together, our results indicate that already minor changes in the hinge design of TbsAbs can have significant impact on the anti-tumor activity of TbsAbs and may provide a new means to improve their potency.

## 1. Introduction

T-cell engager antibodies (TCEs) redirect cytotoxic T-cells to tumor cells by simultaneously binding to a component of the TCR complex (commonly CD3E) and a tumor associated antigen (TAA) on tumor cells [1]. Due to the clinical success of the bispecific T-cell engager (BiTE) blinatumomab, approved by the FDA in 2014 [2,3], the majority of bispecific antibodies (BsAbs) in clinical development are currently TCEs [4]. TCEs can further be classified into two broad classes according to their formats: IgG-like or fragment-based TCEs. Currently, the IgG-like T cell redirecting bispecific antibodies (TbsAbs) being the most widely used form, largely due to their longer in vivo serum half-life due to the presence of an Fc region [5].

Although the concept of BsAbs has a long history, due to challenges in BsAb manufacturing, they only began to stimulate the interest of pharmaceutical companies in the past decade. The production of IgG-like BsAbs requires the correct assembly of antibody’s light and heavy chain fragments. A random assembly of four distinctive polypeptide chains may result in 16 combinations [6]. Therefore, in order to manufacture IgG-like BsAb that can reliably be assembled, it is required to ensure the selective formation of the heterodimerized heavy chains (HCs) and the proper pairing of the light chains of each arm with the cognate HC [7]. Multiple recombinant technologies have been developed to ensure the correct formation of IgG-like bispecific antibodies. In our previous study, we developed a novel Fab x sdAb-Fc format which combined a single domain antibody (sdAb) with a conventional antigen-binding fragment (Fab) [8]. Both arms were linked to an Fc domain optimized for heavy-heavy chain heterodimerization by the introduction of matched amino acid mutations, thus ensuring both correct heavy-chain and heavy-light chain assembly [8]. However, the hinge between sdAb and Fc can be designed in various ways, dependent on different applications. Previous studies demonstrated a direct role of the distance between TAA and CD3E binding sites of TbsAbs on T-cell mediated tumor cell lysis [9,10]. In these applications, the authors modulated their format using various approaches with the common objective of shortening the distance between the two arms of TbsAbs which resulted in improved tumor cell lysis [11,12,13,14]. Additional studies looked at the correlation between the length of effector/target cell synapse distance and T-cell mediated tumor killing by alternative strategies, such as tumor antigen epitope distance to the cancer cell membrane or the overall size of the antigen, which can increase the distance between the effector and target cell [9]. Thereby, it has become apparent that TbsAbs that bind to membrane-distal epitopes extend the intermembrane spacing resulting in decreased tumor killing compared to TbsAbs that bind membrane-proximal epitopes [15,16,17,18,19]. Additionally, the size of the targeted antigen can also effectively increase the distance within the synapse between the T-cell and target cell and has been shown to affect TbsAb potency [15,16]. In particular, it was noticed that the IgG hinge region in different IgG subclasses was a major modulator of antibody function. IgG3 molecules have an extended hinge region of 62 amino acids. This long hinge provides superior flexibility and leads to improved phagocytosis. In contrast, other IgG molecules have shorter and less flexible hinge regions, which was associated with improved antibody-dependent cellular cytotoxicity [20]. These findings suggested that the size of the hinge between the heavy chain and the Fab arm may determine the flexibility of the antibody and therefore the cytotoxic effector functions of it. Therefore, we hypothesized that TbsAbs in the Fab x sdAb-Fc format may benefit from a short hinge design.

To address this hypothesis, we constructed and evaluated TbsAbs targeting mouse EGFR and mouse CD3E with two different hinge region lengths connecting the mEGFR binding domain and its cognate constant region. The longer hinge TbsAb (TbsAb.long) format was designed to mimic the distance between two binding sites of conventional IgG format TbsAb, while the shorter hinge TbsAb (TbsAb.short) was designed to minimize the distance between two binding sites. Our results demonstrated that the efficiency of T cell redirected tumor cell killing directly correlated with the proximity of mEGFR and mCD3E binding regions in Fab x sdAb-Fc TbsAbs.

## 2. Results

### 2.1. Designing and Preparation of mCD3E x mEGFR TbsAbs with Different Hinges

**Figure 1:**
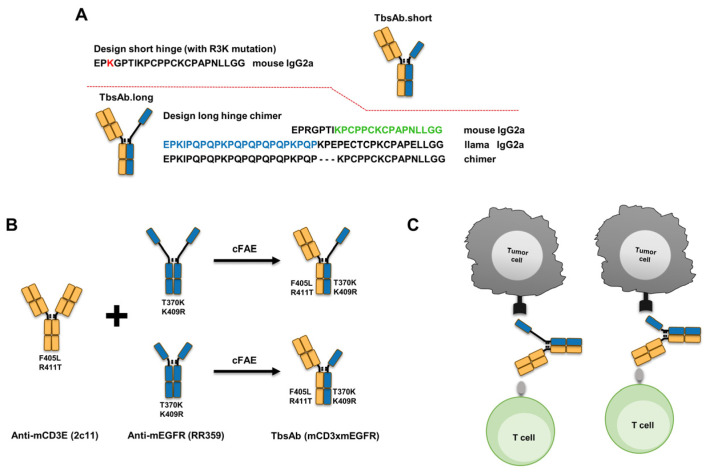
Preparation of Fab x sdAb-Fc TbsAbs with different hinge designs. (**A**) Schematic diagrams of two types of Fab x sdAb-Fc TbsAbs: TbsAb.long, TbsAb.short. (**B**) Schematic illustration of mCD3E × mEGFR TbsAbs generated by the duobody platform. (**C**) Schematic diagrams of tumor cell eliminated by mCD3E × mEGFR TbsAbs.

In order to avoid potential heavy-light chain mispairing during BsAb expression, we previously suggested a novel Fab x sdAb-Fc bispecific antibody format [8]. To further investigate this novel antibody format, we designed and expressed TbsAbs, using a mCD3E Fab-Fc combined with an mEGFR sdAb-Fc with two different hinges. The design of the shorter hinge (23 amino acids in total) was based on the natural mouse IgG2a hinge sequence, with the exception of an Arginine residue at position 3 which we replaced with a lysine residue in order to stabilize the sdAb [21]. The longer hinge (39 amino acids in total) was designed as a chimer hinge based on a combination between a mouse IgG2a hinge and a part of the llama hinge. This resulted in 16 additional amino acids compared with the shorter hinge and mimicked the length of an entire CH1 domain (starts at the end of the hinge and ends with VDKKI, approximately 32.6 Å, Figure S2) [22], in this way, extending the length of the sdAb arm to a similar length of a conventional Fab (Figure 1A). 

To abrogate Fc-FcR mediated effector functions without affecting affinity, LALAPG mutations were introduced to each parental antibody (anti-mCD3E, clone 2c11; anti-mEGFR, clone RR359) [23]. The mCD3E x mEGFR TbsAb with a long hinge (TbsAb.long) and mCD3E x mEGFR TbsAb with a short hinge (TbsAb.short) were then constructed by performing controlled Fab-arm exchange (cFAE) based on the duobody platform (Figure 1B,C). 

**Figure 2:**
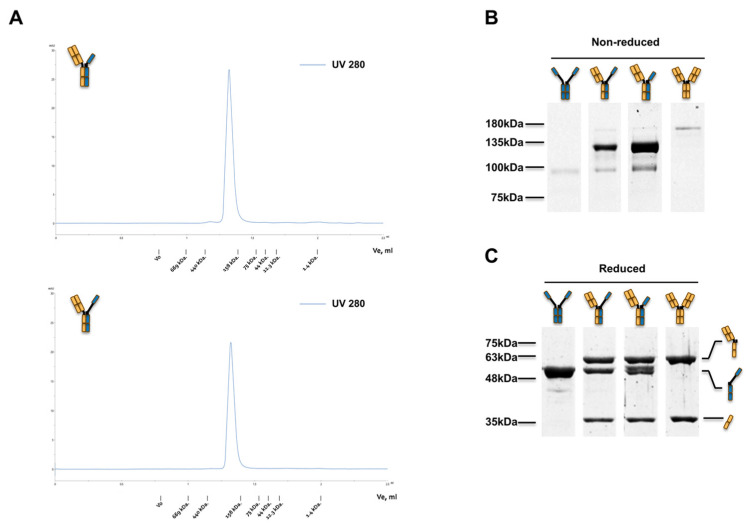
Analysis of the expressed mCD3E × mEGFR TbsAbs. (**A**) Size exclusion chromatography (SEC) analysis of TbsAb.long and TbsAb.short proteins. Ten µL of each respective sample (TbsAb.long and TbsAb.short), at 0.5 mg/mL were eluted by DPBS buffer at a flow rate of 50 µL·min^−1^. (**B**,**C**) SDS-PAGE analysis of TbsAb.long and TbsAb.short proteins under non-reducing and reducing conditions, respectively.

The purity of each expressed TbsAb was analyzed by size-exclusion chromatography (SEC) and sodium dodecyl sulfate–polyacrylamide gel electrophoresis (SDS-PAGE). The monomericity of each parental antibody was >97% (HcAb.RR359.long), >97% (HcAb.RR359.short), and >98% (2c11), respectively (Figure S1). Following cFAE, a single peak was observed in the resulting SEC for each TbsAb and monomericity evaluated was ≥96% (TbsAb.long) and ≥99% (TbsAb.short), respectively (Figure 2A). Under non-reducing SDS-PAGE conditions, the desired TbsAbs showed a predominant band with an MW of ~125 kDa, whereas parental antibodies showed predominant bands with an MW of ~95 kDa or ~165 kDa, respectively (Figure 2B). For TbsAb.long and TbsAb.short, additional minor bands were detected at the same size as of parental antibodies, which indicated minor contamination of the parental mEGFR HcAb and mCD3E mAb in the TbsAb. The purity of TbsAb.long and TbsAb.short were evaluated as ~80.7% and 82.8%, respectively. Under reducing conditions, one band for mEGFR HcAb, two bands for mCD3E mAb and three bands for TbsAbs were detected, as expected (Figure 2C). The bands just below 63 kDa detected in TbsAbs and mCD3E mAb represent the heavy chain of the mCD3E mAb, while the MW bands just above 48 kDa which are detected in mEGFR HcAb and TbsAbs represent the heavy chain of the mEGFR HcAb. The bands at ~35 kDa detected in TbsAbs and mCD3E mAb represent the light chain of the mCD3E mAb. Taken together, these results demonstrate the successful generation of TbsAbs with long and short hinges.

### 2.2. Generation of TbsAb Negative Control Antibody

**Figure 3:**
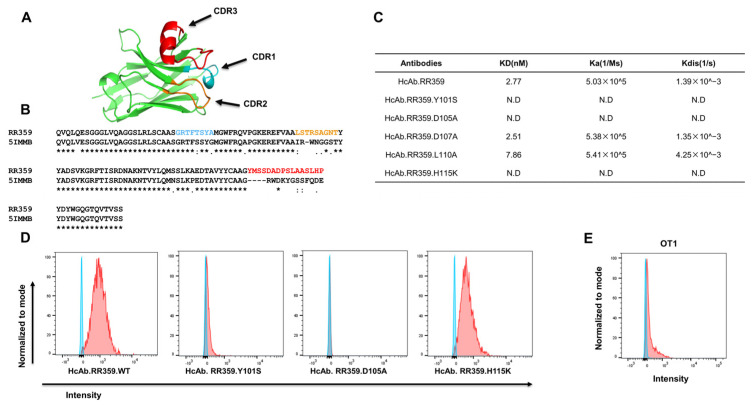
Generation of bispecific negative control antibody by CDR3 mutagenesis. (**A**) Structure of anti-mEGFR sdAb (RR359) predicted by ColabFold. (**B**) The sequence of anti-mEGFR sdAb (RR359) aligned to sdAb (5IMMB). The sequence of CDR1 (blue), CDR2 (orange), CDR3 (red) was indicated. (**C**) The affinity of mutated anti-mEGFR HcAbs measured by Octet. (**D**) The binding of mutated mEGFR HcAbs to CHO/mEGFR cell line. (**E**) TbsAb.con (mCD3E x mEGFR.D105A) binds to OT-1 cells detected by flow cytometry. N.D, not detectable. The “*” (asterisk) indicates positions with conserved residues. The “:” (colon) indicates conservation between groups of amino acids with similar properties. The “.” (period) indicates amino acids with weakly similar properties.

In order to express an appropriate negative control TbsAb [24], we disrupted the binding ability of the mEGFR arm of the TbsAb to mEGFR through site-directed mutagenesis. We started out by evaluating the structure of mEGFR sdAb (RR359) using ColabFold (Figure 3A and Figure S3) [25]. By aligning the sequence of mEGFR sdAb to the PDB database, the molecule with PDB ID 5IMMB was found to be the most similar sdAb to mEGFR sdAb, which facilitated the identification of CDR1,2,3 of mEGFR sdAb (Figure 3B). Given that the CDR3 of the mEGFR sdAb differs most from 5IMMB, we considered it was very likely the region that determined the antibody specificity. Consequently, we performed targeted mutagenesis on this region. Based on the computational analysis of the interactions between sdAb and mEGFR protein by using Discovery Studio software, several amino acids (e.g., Y101, D105, D107, L110, H115 etc.) appeared to be critical for antigen binding. A set of HcAbs containing site-specific mutations were expressed and subsequently affinity to mEGFR recombinant protein was measured by biolayer interferometry using Octet (Figure S4). The HcAbs with Y101S, D105A or H115K mutation showed no detectable binding to mEGFR protein based on the Octet results (Figure 3C). Considering the mEGFR recombinant protein might differ in structure from the natural mEGFR, the binding activity of the HcAbs with either Y101S, D105A or H115K mutation were further examined by FACS, using a CHO cell line overexpressing the mEGFR. No binding of the HcAb with D105A mutation in CDR3 was confirmed with CHO/mEGFR cells (Figure 3D). This version of HcAb was subsequently incorporated into a mCD3E × mEGFR TbsAb by cFAE and used as negative control bispecific antibody (TbsAb.con). The expressed TbsAb.con showed similar purity to the other TbsAbs (Figure S5) and retained its binding capacity to mCD3E (Figure 3E).

### 2.3. mCD3E x mEGFR TbsAb.short Molecule Mediated Enhanced T Cell Redirected Killing In Vitro

**Figure 4:**
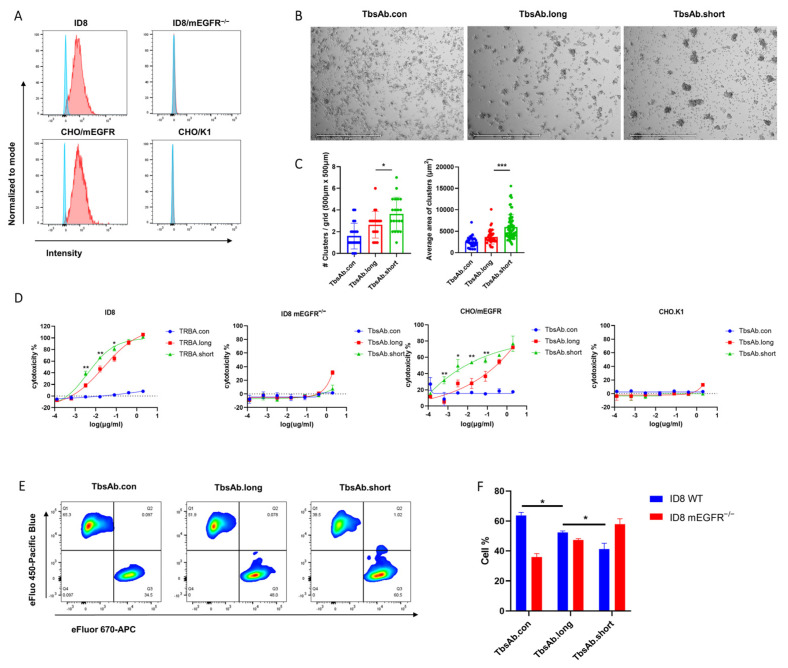
The TbsAb.short form mediates superior T cell mediated cytotoxic. (**A**) Expression of mEGFR on ID8, ID8/mEGFR^−^^/^^−^, CHO/mEGFR and CHO/K1 cell lines were measured by flow cytometry. (**B**) OT-1 T cell blasts with CHO/mEGFR cells in the presence of 0.02688 nM (0.0032 μg/mL) of either mCD3E x mEGFR TbsAb.long or TbsAb.short at an E: T ratio of 5:1 following 24 h incubation. Images were obtained under 4 × magnification, and scale bars 650 μm. (**C**) Quantified number of T cell blasts per grid and average area per T cell blasts by EVOS analysis software. (**D**) In vitro cytotoxicity assay of mCD3E × mEGFR TbsAbs using LDH release assay. Curves were fitted using a four-parameter logistic fitting with GraphPad Prism 8. (**E**) In vitro cytotoxicity assay of mCD3E × mEGFR TbsAbs (0.02688 nM, equal to 0.0032 μg/mL) using FACS. (**F**). Data points represent the mean of three samples; error bars, SD. *: *p* < 0.05; **: *p* < 0.01; ***: *p* < 0.001.

To investigate the capacity of the purified TbsAb for mEGFR+ cell killing, Lactate dehydrogenase (LDH) release cytotoxicity assays were performed. As target cells in the cytotoxicity assays, different mEGFR+ cell lines were used. In order to determine mEGFR expression level on target cells, ID8, CHO/mEGFR, and CHO/K1 were stained with RR359 and antibody binding was measured by FACS (Figure 4A). In order to generate an mEGFR-deficient control cell line, CRISPR/Cas9 was performed to disrupt the mEGFR gene on ID8 cells (Figure 4A). As effector T cells, OT-1s were derived from the spleens of OT-1 × *Rag1^−/−^* mice. OT-1 CD8 T-cells were derived from a C57BL/6 mouse strain, transgenic for a T-cell receptor (TCR) which recognizes an immunodominant ovalbumin-derived epitope. These T-cells can be activated with their cognate antigen, the ovalbumin-derived peptide SIINFEKL. Following a 48 h incubation with the SIINFEKL peptide, activated OT-1 s and target cells were mixed and incubated with each TbsAb for 24 h. At the concentration of 0.02688 nM (0.0032 μg/mL) of each group, significantly more and bigger T cell clumps were observed in the TbsAb.short-treated group compared to TbsAb.long and TbsAb.con treated groups, which suggested stronger proliferation (T-cell blast) of T cells induced by the TbsAb.short form than by the other two TbsAbs (Figure 4B,C). Furthermore, the LDH release assay showed higher LDH release in the presence of the TbsAb.short molecules, suggesting that the TbsAb.short form induced better T-cell mediated cytotoxicity towards ID8 and CHO/mEGFR cells than the TbsAb.long form. The TbsAb.con form appeared not to induce any specific cell lysis. In addition, mEGFR negative cell lines, ID8/mEGFR^−/−^ and CHO.K1, showed no specific cell lysis induced in the presence of either the TbsAb.long, the TbsAb.short or the TbsAb.con form (Figure 4D).

To corroborate these findings, an alternative approach was used to measure tumor cell killing. To this end, ID8 cells and ID8 mEGFR^−/−^ cells were labeled by eFluo 450 or eFluor 670, respectively, and incubated with OT-1 cells at a concentration of 0.02688 nM (0.0032 μg/mL) for each TbsAb or PBS for 24 h. OT-1 cells were carefully washed off by PBS and adherent ID8 cells were then harvested using trypsin detachment. Flow cytometry was performed to detect live ID8 and ID8 mEGFR^−/−^ cells (Figure 4E). As shown in Figure 4F, the TbsAb.short form depleted mEGFR-expressing ID8 cells significantly better than the TbsAb.long form (Figure 4F). 

Taken together, using two different approaches, these data strongly suggest that mCD3E × mEGFR TbsAb.short molecules mediate better T cell redirected killing than the TbsAb.long form.

### 2.4. mCD3E × mEGFR TbsAb.short Mediated Enhanced Cell–Cell Association In Vitro

**Figure 5:**
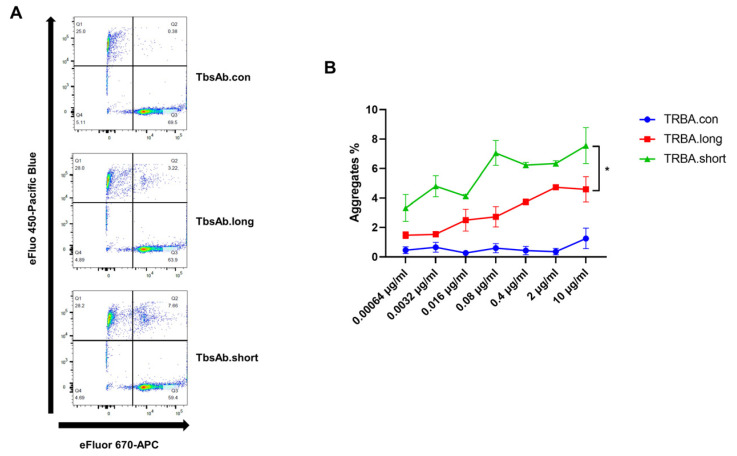
The TbsAb.short form shows better cell aggregate formation than the TbsAb.long form. OT-1 cells were labeled with eFlour 670 dye, and ID8 cells were labeled with the eFlour 450 dye. (**A**) Cells were incubated for 30 min at room temperature with TbsAb.con, TbsAb.long or TbsAb.short molecules at 0.672 nM (0.08 μg/mL). The OT-1-ID8 cell-cell association was determined using flow cytometry and quantified as the percentage of eFlour 450 and eFlour 670 double positive cells in the upper right quadrant. (**B**) The experiment was repeated using increasing concentrations for each of the molecules. Each experimental point was set up in duplicate and the mean SD was plotted. *: *p* < 0.05.

To investigate the underlying mechanisms, which may lead to the improved redirected T cell killing observed with the mCD3E × mEGFR TbsAb.short molecules, we evaluated the ability of the different TbsAbs to induce ID8/OT-1 cell association. Non-activated, naïve OT-1 cells and ID8 cells were used for association and cell aggregation, as measured by FACS. ID8 and OT-1 cells were stained with two different cell-staining dyes and subsequently mixed and incubated with TbsAb.long, TbsAb.short or TbsAb.con. The flow cytometry results showed that a clear cell–cell association was observed with both TbsAb.long and TbsAb.short molecules, but not with the TbsAb.con control construct (Figure 5A,B). Furthermore, the TbsAb.short molecules could induce more cell aggregates than the TbsAb.long form (up to ~8% of the total cell population in the presence of TbsAb.short molecules compared to ~4% of the cell aggregates in the presence of the TbsAb.long molecules). Such an enhanced level of cell–cell association mediated by TbsAb.short molecules was observed consistently across a range of concentrations of the bispecific antibodies. As expected, the TbsAb.con molecule did not induce the cell–cell association of ID8 and OT-1 cells at any concentration tested (Figure 5B). Taken together, these data show that the TbsAb.short form has a higher capacity to form cell aggregates than the TbsAb.long form. Compared to ~30% cell aggregates we reported in our previous study and ~5–17% cell aggregates that others have typically reported [8,18], the relatively lower percentage of cell aggregates induced in our experiments might be for several reasons. So far, we have not yet followed up on these, nevertheless, several aspects such as the expression level of antigens, the affinity of antibodies used or the geometry of the specific antigens could all influence the efficiency with which such TbsAb molecules can link two different cell populations.

### 2.5. mCD3E × mEGFR TbsAb.short Mediated Enhanced T Cell Activation In Vitro

**Figure 6:**
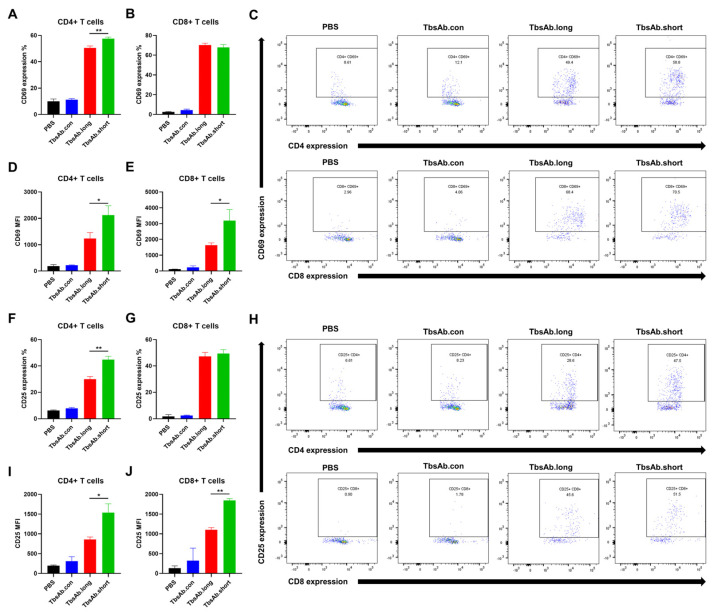
The TbsAb.short form induces superior T cell activation. (**A**–**E**) The expression level of CD69 on CD4+ and CD8+ T cells was detected after incubating with each TbsAb for 24 h (E:T = 5:1). (**F**–**J**) The expression level of CD25 on CD4+ and CD8+ T cells was detected after incubating with each TbsAb for 24 h (E:T = 5:1). *: *p* < 0.05; **: *p* < 0.01.

To investigate to what extent either form of the mCD3E × mEGFR TbsAb could induce T cell activation, splenocytes were mixed with ID8 cells in the presence or absence of the different TbsAb forms and the expression levels of the early activation marker CD69 and of the late activation marker CD25 were determined by flow cytometry on CD4+ and CD8+ T cells after 24 h of in vitro incubation. Our data show that in the presence of ID8 cells, the expression levels of activation marker CD69 and CD25 were considerably upregulated by the TbsAb.long form as well as by the TbsAb.short form. However, in comparison to the TbsAb.long form, the TbsAb.short form activated a significantly higher fraction of CD4 T cells (Figure 6A,F). Furthermore, on a single cell level, the TbsAb.short form activated a significantly higher expression levels of CD69 and CD25 per cell (Figure 6D,I). For CD8 T cells, both TbsAb forms activated a similar fraction of CD8 T-cells (Figure 6B,G), but the TbsAb.short form induced significantly higher expression levels of CD69 and CD25 than the TbsAb.long form (Figure 6E,J). Gating strategy is shown in Figure S6. These data strongly suggest that while both TbsAb can induce T cell activation, the TbsAb with the shorter hinge induced a stronger T cell activation than the TbsAb with the longer hinge.

## 3. Discussion

Several different factors have been described that can affect the potency of bispecific T-cell engagers. These include the copy number of the targeted antigen, the size of the antigen, and the distance of the target epitope to the membrane, as well as antibody formats with different sizes, valences, and geometries [9,10]. In this study, we focused on modulating the distance between T cells and tumor cells, by modulating the hinge region between the heavy chain backbone of the antibody molecule and the Fab arm. To this end, two mCD3E × mEGFR bispecific antibodies with different hinge designs in the Fab x sdAb-Fc format were generated; one with a shorter hinge and one with a longer hinge design. As our results show, the TbsAb.short molecule exhibited significantly greater potency than the TbsAb.long molecule in T-cell redirected killing (Figure 4D). Furthermore, the TbsAb.short form linked T cells to mEGFR-expressing tumor cells more efficiently than the TbsAb.long form by forming more T cell-tumor cell aggregates. In addition, as measured by CD69 and CD25 expression, T cells appeared to be more strongly activated by the TbsAb.short than by the TbsAb.long format. 

Since the same antigen recognition specificities were used, the enhancement observed with the TbsAb.short molecule was presumably due to the hinge difference in the molecules. Such a finding is consistent with previous studies. Bluemel and colleagues designed TbsAbs to target different epitopes on human melanoma chondroitin sulfate proteoglycan (hMCSP) and found that the distance of the binding domain to the target cell membrane had a significant impact on the potency of T cell redirecting bispecific antibodies [16]. Additionally, several more recent studies, targeting other TAAs such as FcRH5, ROR1, and CD3E, further confirmed that targeting membrane-proximal epitopes, which also shortened the distance between T cells and tumor cells, could improve the in vitro potency of their antibody constructs to facilitate T cell mediated target cell lysis [15,19,26]. In yet another study, TbsAbs in conventional IgG2 format were compared to a Diabody-Fc (DbFc) format in in vitro tumor cell cytotoxicity assays. The DbFc format shortened the distance between antigen-binding arms and turned out to be more potent [27]. With TbsAbAs in all these cases, also in the one we have described here, several factors may contribute to the enhanced tumor elimination potency by using a short hinge format. For instance, we found that the TbsAb.short molecule induced significantly more T cell-tumor cell aggregation than the TbsAb.long molecule (Figure 5). Such improved aggregation could facilitate the T cell mediated attack of tumor cells. It has been reported that immunological synapse formation requires an optimal distance between T cells and tumor cells [10]. This optimal distance might be influenced by the geometric configurations of the different antibody constructs. In line with such an assumption, it was reported previously, that targeting membrane-proximal epitope by TbsAbs could facilitate efficient T cell synapse formation leading to enhanced tumor cell elimination [15]. Based on this finding, one could argue that possibly by forming tight immunological synapses the TbsAb.short form might be more potent in T cell redirected tumor cell elimination than the TbsAb.long form. Alternatively, efficient T cell-tumor cell engagement might form multiple TbsAbs mediated connections at the immune synapse. In such a situation, the property of individual TbsAbs, such as the flexibility of the binding sites, could be an important factor in forming efficient T cell-tumor cell aggregation [14,15]. In line with such an assumption, it has been reported by Kapelskia et al. that the different flexibility shown in the hinge region of human IgG subclasses (IgG1 > IgG4 > IgG2, IgG1 being the most flexible) significantly impacted the T cell redirected tumor cell elimination [28]. Compared to the TbsAb.long molecule, the TbsAb.short molecule has a shorter and relatively rigid hinge, which results in a relatively fixed distance and orientation between the two binding domains. Consequently, given the same specific concentration of each TbsAb, the binding arm of randomly free-floating TbsAb.long molecules would require more time to adjust the preferable orientation to bridging T cells and tumor cells than the more rigid TbsAb.short molecules. In addition, due to the dynamic and reversible binding property of TbsAbs, once the T cell-tumor cell aggregation has been formed, TbsAb.short could keep the distance between T cells and tumor cells shorter. This might facilitate other free TbsAb.short molecules to support and further enhance this originally brief interaction between T cells and tumor cells; consequently, resulting in a tighter T cell-tumor cell aggregation than the TbsAb.long molecule might be able to establish. In line with such an assumption, the TbsAb.short construct induced in comparison to the TbsAb.long construct increased CD69 and CD25 expression on T cells, suggesting a more extensive activation of the T cells. T cell activation is generally considered to be sensitive to consistent TCR signaling, which is triggered by constant antigen/receptor interaction [29]. Therefore, the higher expression level of CD69 and CD25 could be assumed to be a direct result of a tighter T cell: tumor cell aggregation induced and maintained by the TbsAb.short molecule. 

In summary, we investigated the potential application of the Fab x sdAb-Fc bispecific format for T-cell mediated tumor cell killing. We demonstrated that a TbsAb with a shorter distance between the two arms allows for a tighter bridging between the tumor cells and the effector T cells, subsequently leading to a more robust T cell activation and in turn greater tumor cell killing. Instead of comparing different bispecific formats [14,30,31,32,33,34,35,36], here, we demonstrated that by already changing a dozen of the amino acids in the hinge region in the same bispecific format could induce a substantial impact on its cytotoxic activity. So far, our data have been limited to mouse antibody constructs. However, the hinge length of human antibodies differs from that of mouse antibodies. Therefore, additional research might be needed to apply our findings to human antibody constructs. Nonetheless, our data strongly suggest that the development of human Fab x sdAb-Fc TbsAb for the treatment of cancers could potentially benefit from a shortened hinge design. Therefore, our data indicate that the modulation of the ‘hinge region length’ parameter could potentially also be applied to other IgG-like TbsAb formats in order to optimize their efficacy. 

## 4. Materials and Methods

### 4.1. Cell Lines

CHO.K1 and CHO/mEGFR cell lines were described in our previous study [8]. Mouse ovarian cancer surface epithelial cell line (ID8) was kindly provided by Professor Rose Zamoyska, the cells were cultured in IMDM (ThermoFisher Scientific, UK #I3390-500ML) supplemented with 10% FBS (Gibco, UK #10500-064), 1% L-glutamine (ThermoFisher Scientific, UK #25030-081), 0.1% 2-mercaptoethanol (Sigma-Aldrich) in 10-cm-Petri dishes and incubated at 37 °C. ID8/mEGFR^−/−^ cell line was generated by CRISP/Cas9 gene-editing system. Cloning was performed using Truecut V2 cas9 (ThermoFisher Scientific). ID8 cells were transfected with CRISPR plasmid-containing gRNA for mEGFR-KO by electroporation (1600V, 10ms, 3 pulses) using the InvitrogenTM NeonTM Transfection System (ThermoFisher Scientific). TRACR RNA was obtained from Integrated DNA technologies and the gRNA sequence is 5′ CCTCATTGCCCTCAACACCG 3′. The transfected cells were cultured with IMDM (ThermoFisher Scientific, UK #I3390-500ML) for 4 days before sorting. Cells were stained with HcAb anti-mouse EGFR (clone RR359), followed by anti-mouse IgG2a-PE (1:100). Using the BD FACSAriaTM II sorter, the mEGFR- ID8 population was bulk-sorted based on mEGFR expression. Sorted ID8 clones without mEGFR expression were isolated and expanded.

OT-1 cells were derived from spleens of OT-1 Rag1^−/−^ (C57BL/6) mice. Spleens were gently dissociated mechanically through a 70 μm filter. The suspension was then centrifuged at 300× *g* at 4 °C for 5 min, the supernatant discarded, and RBC lysis buffer (Sigma-Aldrich, UK #R7757-100ML) was added. The suspension was incubated for 5 min at room temperature, cells were then centrifuged again and the pellet was resuspended in IMDM. Cells were counted and cultured in IMDM (ThermoFisher Scientific, UK #I3390-500ML) supplemented with 10% FBS (Gibco, UK #10500-064), 1% L-glutamine (ThermoFisher Scientific, UK #25030-081), 0.1% 2-mercaptoethanol (Sigma-Aldrich, USA #M6250) in 10 cm petri dishes and incubated at 37 °C.

### 4.2. Designation and Construction of Expression Vectors for Bispecific Antibodies

By using the established duobody platform, we designed IgG2a-mCD3E × mEGFR TbsAbs in a Fab × sdAb-Fc format previously developed by Huang et al. with two different hinge lengths. These comprised (i) the variable light chain (VL) and variable heavy chain (VH) domains of 2c11, an anti-mCD3E monoclonal antibody, (ii) VH domain of anti-mEGFR single domain antibody RR359, and (iii) a mouse IgG2a Fc module with duobody mutations for heterodimerization.

The anti-mEGFR sdAb (RR359) was described previously and the amino acid sequence of anti-mCD3E (2c11) was obtained from the IMGT database [8,37]. The amino acid mutations introduced to each vector to allow heavy-heavy chain heterodimerization are depicted in Figure 3C. T370K and K409R point mutations were introduced to the CH3 region of heavy chain only anti-mEGFR antibody RR359. F405L and R411T point mutations were introduced to the CH3 region of the conventional anti-mCD3E antibody 2c11. In addition, L234A, L235A, and P329G (LALA-PG) mutations were introduced to the Fc domain of each 2c11 and RR359 sequence to silence their Fc-mediated effector functions. The amino acid sequences of the expressed constructs are shown in supplementary Table S1. The parental mAb expression vectors were constructed by de novo synthesis (GeneArt, ThermoFisher Scientific).

Two different hinge constructs were produced by introducing different linkers in the parental anti-mEGFR plasmids. For the short hinge design, a full mouse IgG2a linker was introduced (EPKGPTIKPCPPCKCPAPNLLGG) between the sdAb part and the Fc part of RR359 antibody. For the long hinge design, a camelid/mouse chimeric linker (EPKIPQPQPKPQPQPQPQPKPQPKPCPPCKCPAPNLLGG) was introduced between the sdAb part and Fc domain of the RR359 antibody (Figure 1). The expression vectors of these antibodies were constructed by de novo synthesis (GeneArt, ThermoFisher Scientific Scientific).

### 4.3. Generation of Bispecific Antibodies

FreeStyleTM 293-F cells (Invitrogen, UK # R79007) were grown in FreeStyle 293 Expression medium (Invitrogen, UK #12338-018). Each relevant heavy and light chain expression vector was co-transfected into FreeStyle™ 293-F cells (Invitrogen, UK # R79007), using 293fectin™ reagent (Invitrogen, UK #12347-019) according to the manufacturer’s recommended conditions. At 7-days post-transfection, the antibodies were purified by protein A affinity chromatography (Peptide Synthetics), dialyzed overnight to PBS (Gibco, UK #D8537-500ML), and filter-sterilized over 0.22-μm filters. Antibody concentration was calculated based on the Beer–Lambert Law, A = ε ×b × c, (A is the A280 absorbance, b is the path length, c is the analyte concentration, and ε is the wavelength-dependent molar absorptivity coefficient with units of M^−1^ cm^−1^). A280 absorbance was measured by spectrophotometry using a Nanodrop ND-1000 system (ThermoFisher Scientific). Equimolar amounts of relevant parental antibodies were mixed and incubated with 2-mercaptoethylamine (2-MEA; Sigma, Switzerland #30078-25G) at a final concentration of 1 mg/mL total antibody in PBS (Gibco, UK #D8537-500ML). The final concentration of 2-MEA was 75 mM. The mixtures were incubated for 5 h at 31 °C. The mixtures were then buffer-exchanged against PBS using Slide-A-Lyzer cassettes (ThermoFisher Scientific, USA #66380) to remove 2-MEA. Samples were stored overnight at 4 °C to allow for the re-oxidation of the disulfide bonds. Bispecific antibody concentration was calculated as previously described. The purity of TbsAbs was evaluated by SDS-PAGE in reducing and non-reducing conditions. 

### 4.4. Generation of Control TbsAb by Mutagenesis

The three-dimensional structure model of mEGFR sdAb was predicted by ColabFold, which combines a protein homolog search MMseqs2 with AlphaFold2 (https://colab.research.google.com/github/sokrypton/ColabFold/blob/main/AlphaFold2.ipynb, accessed on 1 June 2021). The mutations were introduced into the mEGFR binding CDR3 of the RR359.short antibody sequence by site-directed mutagenesis by PCR. mEGFR-His recombinant protein (R&D systems) was diluted to 50 mM in 10 mM acetate pH 5.0 (ForteBio, USA #18-1069) and loaded on NHS/EDC activated AR2G biosensors (ForteBio, USA #18-5088). HcAb.mEGFR antibodies with different mutations were diluted to 20 μg/mL in 10× kinetic buffer (ForteBio, USA #18-1092) and associated to mEGFR-His protein and 10 × kinetic buffer was used as negative control. Binding kinetics were measured by the Octet system according to the manufacturer’s instructions (ForteBio). Data was analyzed using data analysis software HT V10.0 (ForteBio). Signal of negative control was subtracted in the BLI experiment. cFAE was performed as described above, using parental antibodies HcAb.mEGFR with short hinge and D105A mutation and mCD3E mAb to generate the TbsAb.con.

### 4.5. Size-Exclusion Chromatography (HP-SEC)

Aggregation and degradation of TbsAbs were quantified by SEC. Ten µL of each respective sample (TbsAb.long and TbsAb.short), at 0.5 mg/mL were loaded (20 µL load volume) onto a calibrated Superdex-200 Increase 3.2/300 GL (Cytiva, UK #28990946) size exclusion column pre-equilibrated in PBS, pH 7.4, at 8 °C and run with a flow rate of 50 µL·min^−1^ on an ÄKTA PURE Micro™ LC system (Cytiva, UK # 29302479). Elution was monitored at 220 nm, 256 nm, and 280 nm, with 2.5 s integration. The concentration of protein in respective peaks was calculated using the peak analysis software (with a morphological baseline with a skim value of 7.0) provided with the instrument (UNICORN v7.7™; Cytiva) and the relative purity was calculated as a percentage of all integrated peaks.

### 4.6. In Vitro Cytotoxicity Assays

Naïve OT-1 cells (enriched from spleens of OT-1 Rag1^−/−^ mice) were activated by exposure to ovalbumin peptide SIINFEKL (2 ng/mL, Peptide Synthetics) for 48 h. LDH method: Target cells were seeded in IMDM (ThermoFisher Scientific, UK #I3390-500ML) with 10% FBS (ThermoFisher Scientific, UK #10500-064) at a density of 1 × 10^4^ cells/well on a 96-well flat-bottom cell culture plate. Five-fold serial gradient dilution of either TbsAb.long, TbsAb.short, or TbsAb.con was performed in a complete medium, starting with a 16.8 nM (2 µg/mL) concentration and incubated for 0.5 h. Samples were added to corresponding wells at a final volume of 150 μL. Subsequently, in IMDM with 10% inactivated FBS medium, OT-1 cells were adjusted to 5 × 104 cells/well added into the plate at an effector cell:tumor cell (E:T) ratio of 5:1. The cytotoxicity assay was detected after plates were incubated at 37 °C for 24 h from supernatant samples using CytoTox96^®^ Non-Radioactive LDH Kit (Promega, USA #G1781). The cytotoxicity percentages were calculated following the manufacturer’s instructions as shown here:

$$Cytotoxicity\;\%=\frac{signal\;\left(Experimental-Effector\;spontaneous\right)}{signal\;\left(Target\;maximum\right)-signal\left(target\;spontaneous\right)}\times 100\%$$

Flow cytometry method: ID8 cells and ID8 mEGFR^−/−^ cells were labeled by eFluo 450 or eFluor 670, respectively. ID8 cells and ID8 mEGFR^−/−^ were seeded in IMDM (ThermoFisher Scientific, UK #I3390-500ML) with 10% FBS (ThermoFisher Scientific, UK #10500-064) at a density of 2 × 10^4^ cells/well on a 96-well flat-bottom cell culture plate. Five-fold serial gradient dilution of either TbsAb.long, TbsAb.short, or TbsAb.con was performed in a complete medium, starting with a 16.8 nM (2 μg/mL) concentration and incubated for 0.5 h. PBS treated samples were used as negative control. Samples were added to corresponding wells at a final volume of 150 μL. Subsequently, in IMDM with 10% inactivated FBS medium, OT-1 cells were adjusted to 1 × 105 cells/well added into the plate at an effector cell:tumor cell (E:T) ratio of 5:1. The OT-1 cells were washed off by PBS after 24 h incubation. Remaining attached ID8 and ID8 mEGFR^−/−^ cells were detected by flow cytometry. The ID8 lysis percentages were calculated following the formula as shown here:

$$ID8\;lysis\;\%=\left[1-\frac{number\;of\;Pacific\;blue\;positive\;cells\left(TRBA\;treated\right)}{number\;of\;Pacific\;blue\;positive\;cells\left(PBS\;treated\right)}\right]\times 100\%$$

All tests were repeated in triplicates and linear or nonlinear regression analysis to fit dose-response curves were assayed with GraphPad Prism Version 8.0.

### 4.7. Microscopy

Ovalbumin peptide SIINFEKL activated OT-1s were incubated with CHO/mEGFR cells in the presence of TbsAb.con, TbsAb.long or TbsAb.short respectively for 24 h. Images of activated OT-1s were taken with an EVOS M7000 microscope under 4 × magnification. T cell blasts analysis was performed using EVOS analysis software to count the number and calculate the area of T cell blasts in each group. T cell blasts in randomly selected 20 grids (500 μm × 500 μm) of each photo were counted and calculated for analyzing.

### 4.8. Cell-Cell Association Assays

OT-1 cells were labelled with fixable viability dye eFluor 670 (eBiosciences, USA #65-0840-85) as effector cells and ID8 cells were labelled with fixable viability dye eFluor 450 (eBiosciences, USA #65-0842-85) as target cells, according to the manufacturer’s instructions. Mixtures of 5 × 10^4^ cells of each labelled cell line were incubated together at 4 °C for 45 min in a 96-round bottom plate, with 5-fold serially diluted TbsAb.long, TbsAb.short, or TbsAb.con in FACS buffer (PBS supplemented with 1% BSA + 1% EDTA, Gibco), starting with a 84 nM (10 μg/mL) concentration. All tests were repeated in duplicates. Cells were then washed in FACS buffer and resuspended in 200 μL for analysis on a FACSCanto II flow cytometer (BD Biosciences). Data were analyzed using FlowJo v.10.8.0 software (BD Biosciences), and GraphPad Prism Version 9.0 software. Ten thousand events were collected.

### 4.9. T Cell Activation Assays

Freshly isolated splenocytes from the WT C57BL/6 mice (1 × 10^5^ cells/mL) were treated with either TbsAb.long, TbsAb.short or TbsAb.con at 3.36 nM (0.4 μg/mL) and incubated with ID8 target cells (2 × 10^4^ cells/mL) in 96-well plates for 18 h. The splenocytes were collected and stained with CD8-APC (eBioscience, USA #17-0081-83), CD4-Pacific blue (eBioscience, USA, #57004282) and CD69-PE (PharMingen, USA #553237)/CD25-PE (PharMingen, USA #09985B). Cells were counted by flow cytometry on a FACSCanto II system (BD Biosciences) and data were analyzed with FlowJo v.10.8.0 software (BD Biosciences). Percentage of PE positive cells and mean fluorescence intensities (MFI) were used for statistical analysis using GraphPad Prism version 9.0 software.

### 4.10. Statistical Analysis

Statistical analyses were performed using Prism software version 9.0 (GraphPad). P values were determined for comparisons between TbsAb.long and TbsAb.short-mediated T-cell cytotoxicity by paired T-test and T-cell activation by unpaired *t*-test. *p* values for comparisons between TbsAb.long and TbsAb.short-mediated cell-cell association were determined by 2-way ANOVA test. For all statistical tests, results with a *p* value <0.05 were considered significant. *: *p* < 0.05; **: *p* < 0.01; ***: *p* < 0.001.

## Data Availability

Original data sets will be made available upon request.

## Supplementary Materials

The following supporting information can be downloaded at: https://www.mdpi.com/article/10.3390/biom12101331/s1, Figure S1: Monomericity of each parental antibody (RR359.long, RR359.short and 2c11) analyzed by size-exclusion chromatography (SEC); Figure S2: Length of antibody CH1 evaluated by Discovery Studio; Figure S3: Prediction quality judged by visualizing multiple sequence alignments (MSA) depth and showing the AlphaFold2 confidence measures; Figure S4: Raw data binding kinetics of HcAb.RR359 with different mutations to AR2G-sensor bound mEGFR-His protein as measured by BLI on an Octet machine; Figure S5: Purity of TRBA.con evaluated by SDS-Page at non-reducing condition; Figure S6: The gating strategy for the T-cells shown in Figure 6; Table S1: The amino acid sequences of the expressed constructs.
