# Supplementary material for: Shortened Hinge Design of Fab x sdAb-Fc Bispecific Antibodies Enhances Redirected T-Cell Killing of Tumor Cells
Source: Biomolecules. 2022 Sep 21;12(10):1331. doi: 10.3390/biom12101331 (PMC9599842; doi:10.3390/biom12101331)
Supplement: Supplementary file 1 [file biomolecules-12-01331-s001.zip › biomolecules-1908398-supplementary.pdf]

Supplementary Figures:

RR359.long

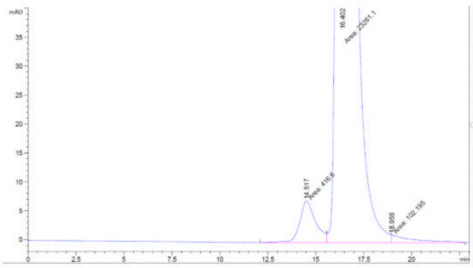

Peak #	RetTime [min]	Type	Width [min]	Area [mAU*s]	Height [mAU]	Area %
1	14.517	MF	0.9661	416.60031	7.18733	1.7519
2	16.402	FM	0.6415	2.32611e4	604.36542	97.8183
3	18.956	FM	1.2788	102.19473	1.33190	0.4298

RR359.short

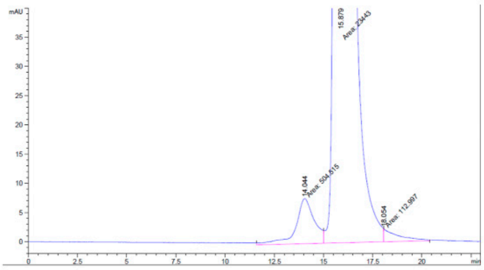

Peak #	RetTime [min]	Type	Width [min]	Area [mAU*s]	Height [mAU]	Area %
1	14.044	MF	1.0872	504.51495	7.73421	2.0969
2	15.879	MF	0.6357	2.34430e4	614.60223	97.4335
3	18.054	FM	0.8328	112.99677	2.26125	0.4696

2c11

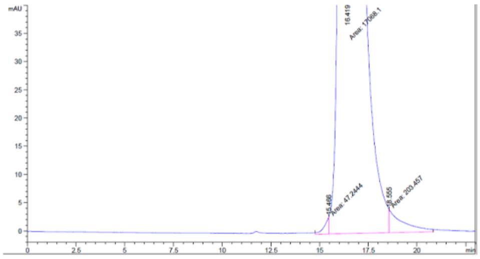

Peak #	RetTime [min]	Type	Width [min]	Area [mAU*s]	Height [mAU]	Area %
1	15.466	MF	0.2598	47.24444	3.03024	0.2728
2	16.419	MF	0.9097	1.70681e4	312.69217	98.5524
3	18.555	FM	0.8125	203.45674	4.17334	1.1748

Figure S1. Monomericity of each parental antibody (RR359.long, RR359.short and 2c11) analyzed by size-exclusion chromatography (SEC).

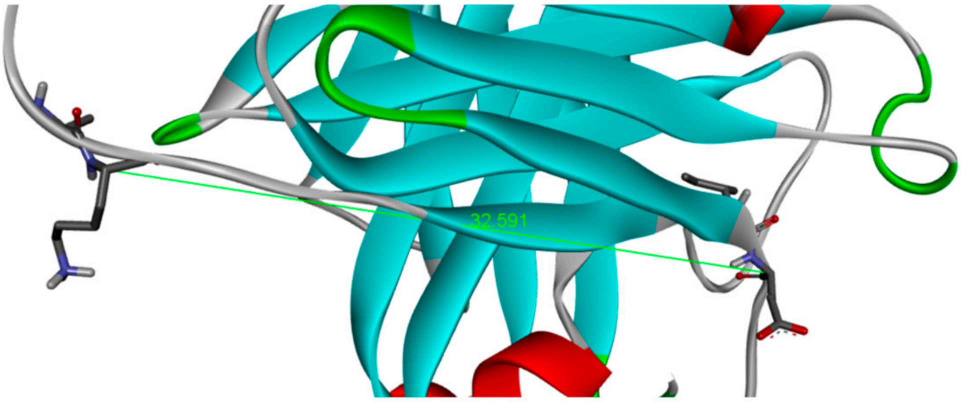

Figure S2. Length of antibody CH1 evaluated by Discovery Studio (used PDB ID 3R06 as template).

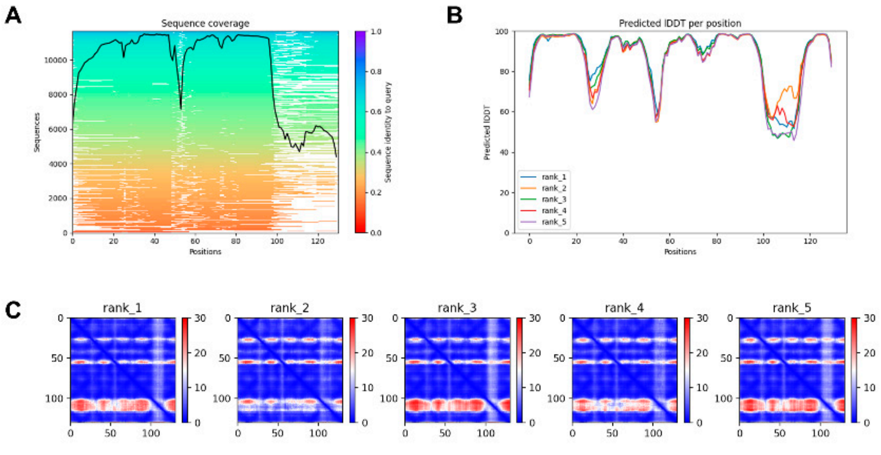

Figure S3. Prediction quality judged by visualizing multiple sequence alignments (MSA) depth and showing the AlphaFold2 confidence measures. (A) Multiple sequence alignments. Number of sequences per position, the higher the better. (B) Predicted local distance difference test (IDDT) per position. Model confidence (out of 100) at each position. The higher the better. (C) Predicted alignment error (PAE). A useful metric to assess how confident the model is about the interface. The lower the better. Rank 1 structure was used in this study.

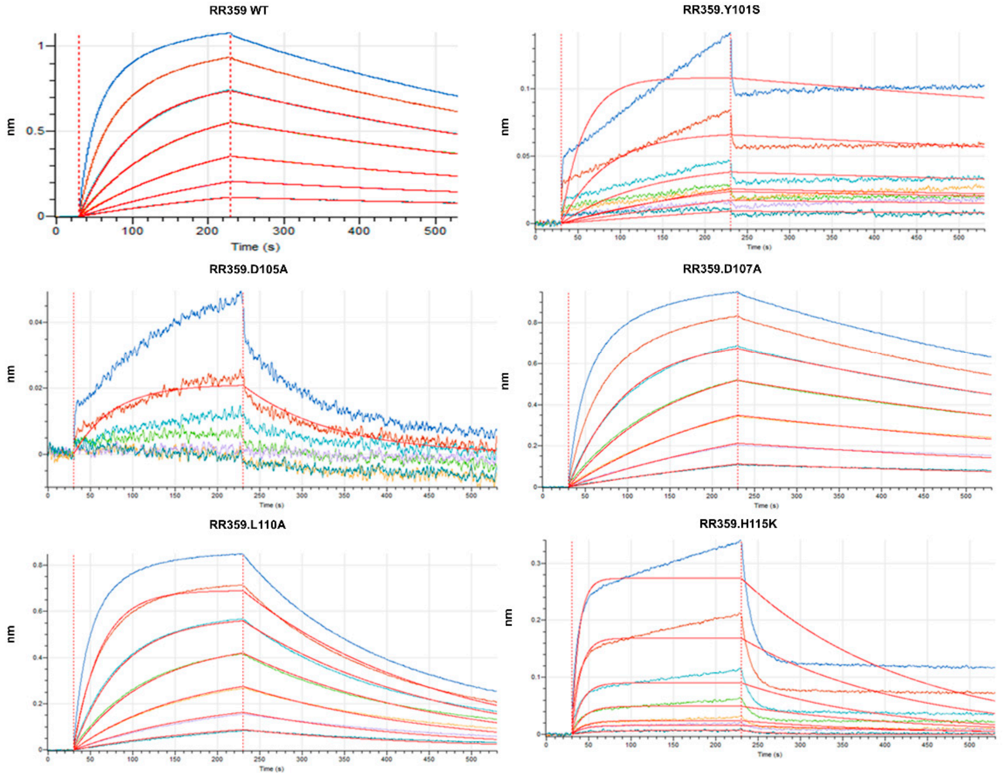

Figure S4. Raw data binding kinetics of HcAb.RR359 with different mutations to AR2G-sensor bound mEGFR-His protein as measured by BLI on an Octet machine.

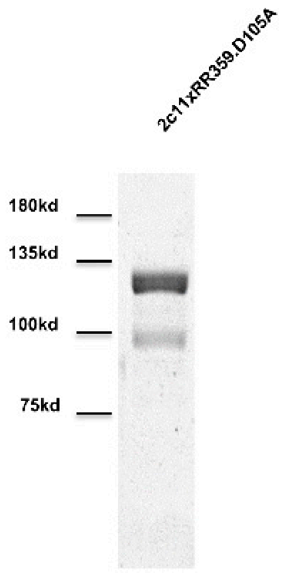

Figure S5. Purity of TbsAb.con evaluated by SDS-Page at non-reducing condition.

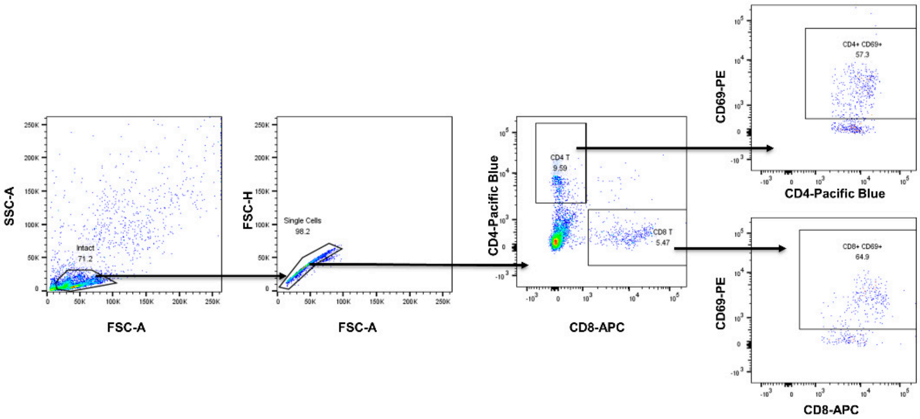

Figure S6. The gating strategy for the T-cells shown in Figure 6.

Supplementary Table S1.

**RR359(anti-mEGFR)-long-migg2a.LALAPG.T370K.K409R**
QVQLQESGGGLVQAGGSLRLSCAASGRT-<br>FTSYAMGWFRQVPGKEREFVAALSTRSAGNTY-<br>YADSVKGRFTISRDNAKNTVYLQMSSLKAEDTAVYYCAAGYMSSDADPSLAASLHPY<br>DYWGQGTQVTVSSEPKIPQPQPKPQPQPQPQPKPQPKPCPPCKCPAP-<br>NAAGGPSVFIFPPKIKDVLMISLSPMVTCVVVDVSEDDPDVQISWFVNNVEVLTAQTQ<br>THREDYNSTLRVVSALPIQHQDWMSGKEFKCKVNNKALGAPIERTI-<br>SKPKGSVRAPQVYVLPPPEEEMTKKQVTLTCMVKDFMPEDIYVEWTNNGKTELNYK<br>NTEPVLDSDGSYFMYSRLRVEKKNWVERNSYSCSVVHEGLHNHHTTKSFSRTPGK
**RR359(anti-mEGFR)-sh-migg2a.LALAPG.T370K.K409R**
QVQLQESGGGLVQAGGSLRLSCAASGRT-<br>FTSYAMGWFRQVPGKEREFVAALSTRSAGNTY-<br>YADSVKGRFTISRDNAKNTVYLQMSSLKAEDTAVYYCAAGYMSSDADPSLAASLHPY<br>DYWGQGTQVTVSSEPKGPTIKPCPPCKCPAPNAAGGPSVFIFPPKIKDVLMIS-<br>LSPMVTCVVVDVSEDDPDVQISWFVNNVEVLTAQTQTHREDYNSTLRVVSALPIQHQ<br>DWMSGKEFKCKVNNKALGAPIERTISKPKGSVRAPQVYVLPP-<br>PEEEMTKKQVTLTCMVKDFMPEDIYVEWTNNGKTELNYKNTEPVLDSDGSYFMYSR<br>LRVEKKNWVERNSYSCSVVHEGLHNHHTTKSFSRTPGK
**RR359(anti-mEGFR)-sh-migg2a.D105A.LALAPG.T370K.K409R**
QVQLQESGGGLVQAGGSLRLSCAASGRT-<br>FTSYAMGWFRQVPGKEREFVAALSTRSAGNTY-<br>YADSVKGRFTISRDNAKNTVYLQMSSLKAEDTAVYYCAAGYMSSAADPSLAASLHPY<br>DYWGQGTQVTVSSEPKGPTIKPCPPCKCPAPNAAGGPSVFIFPPKIKDVLMIS-<br>LSPMVTCVVVDVSEDDPDVQISWFVNNVEVLTAQTQTHREDYNSTLRVVSALPIQHQ<br>DWMSGKEFKCKVNNKALGAPIERTISKPKGSVRAPQVYVLPP-<br>PEEEMTKKQVTLTCMVKDFMPEDIYVEWTNNGKTELNYKNTEPVLDSDGSYFMYSR<br>LRVEKKNWVERNSYSCSVVHEGLHNHHTTKSFSRTPGK
**2c11(anti-mCD3E)-migg2a.LALAPG.F405L.R411T**
EVQLVESGGGLVQPGKSLKLSCEASGFTFSGYGMHWVRQAPGRGLESVAYITSSSINI-<br>KYADAVKGRFTVSRDNAKNLLFLQMNILKSEDTAMYYCARFDWDKNYWGQGTMV<br>TVSSAKTTAPSVYPLAPVCGDTTGSSVTLGCLVKGYFPEPVTLTWNSGSLSSGVHT-<br>FPAVLQSDLYTLSSSVTVTSSTWPSQSITCNVAHPASSTKVDKKIEPRGPTIKPCPPCKCP<br>APNAAGGPSVFIFPPKIKDVLMISLSPMVTCVVVDVSEDDPDVQISWFVNN-<br>VEVLTAQ-<br>TQTHREDYNSTLRVVSALPIQHQDWMSGKEFKCKVNNKALGAPIERTISKPKGSVRA<br>PQVYVLPPPEEEMTKKQVTLTCMVTDFMPEDIYVEWTNNGKTEL-<br>NYKNTEPVLDSDGSYL-<br>MYSKLTVEKKNWVERNSYSCSVVHEGLHNHHTTKSFSRTPGK
**2c11(anti-mCD3E), light chain**
DIQMTQSPSSLPASLGDRVTINCQASQDISNYLNWYQQKPGKAP-<br>KLLIYYTNKLADGVPSRFSGSGSGRDSSFTISSLESEDIGSYYCQQYYNYPWTFGPGTKL<br>EIKRADAAPTVSIFPPSSEQLTSGGASVVCFLNNFYPKDINVKWKIDGS-<br>ERQNGVLNSWTDQDSKDSTYSMSSTLTLTKDEYERHNSYTCEATHKTSTSPIVKSFNR<br>NEC
